# Amygdala neurofeedback as an adjunct to residential dialectical behavior therapy for borderline personality disorder: a pilot randomized controlled trial

**DOI:** 10.1186/s12888-026-08767-7

**Published:** 2026-10-16

**Authors:** Miroslava Hofmanová, Philip S. Santangelo, Nikolaus Kleindienst, Christian Schmahl, Christian Paret

**Affiliations:** 1 Department of Psychosomatic Medicine and Psychotherapy, Central Institute of Mental Health, Medical Faculty Mannheim, University of Heidelberg, Mannheim, Germany; 2 German Center for Mental Health (DZPG), Partner Site Mannheim - Heidelberg - Ulm, Mannheim, Germany; 3 Department of Behavioural and Cognitive Sciences, University of Luxembourg, Esch-sur-Alzette, Luxembourg

**Keywords:** Amygdala, Neurofeedback, Borderline personality disorder, Dialectical beahavior therapy, Ecological momentary assessment, Clinical trial

## Abstract

**Background:**

Borderline personality disorder (BPD) is characterized by affective instability and amygdala hyperreactivity. Although dialectical behavior therapy (DBT) reduces symptoms and amygdala-related abnormalities, many patients show only partial remission. Real-time functional magnetic resonance imaging (fMRI) amygdala neurofeedback (amy-NF) directly targets hyperactive limbic responses and has shown promise in uncontrolled trials. We examined whether amy-NF adds benefit to inpatient DBT.

**Methods:**

Thirty-six mid-therapy people with BPD and clinically significant residual symptoms (BSL-23 > 1.5) were randomized to DBT plus amy-NF (*n* = 23) or DBT alone, termed treatment as usual (TAU = 13). Post-treatment assessments were completed by 15/9 (amy-NF/TAU), with 15/8 and 12/7 completing the 3- and 6-month follow-ups. Ecological momentary assessment (EMA) of daily-life affective instability served as the primary outcome. Secondary outcomes included self-reports, amygdala responses during two fMRI tasks, and resting-state fMRI. Assessments occurred at pre-, post-, 3-, and 6-month follow-up.

**Results:**

Participants successfully downregulated amygdala activity during neurofeedback. EMA showed improvements in negative affect, inner tension, and affective instability over time, without consistent superiority of amy-NF. Self-reports likewise showed no additional benefit of amy-NF, and BSL-23 scores increased after discharge in both groups. Amy-NF was associated with transient pre–post reductions in amygdala activity during both viewing and regulation in the emotion regulation task, and with more stable amygdala reactivity over time than TAU in the faces task. Resting-state analyses showed transient pre–post increases in amygdala connectivity with precuneus and dorsomedial frontal regions in amy-NF.

**Conclusions:**

Amygdala neurofeedback is feasible and reduced amygdala activity in BPD, but did not yield additional clinical benefit when delivered alongside intensive residential DBT, although the small sample may have limited power to detect such effects.

**Trial registration:**

The study protocol was preregistered 2020/03/26 on ClinicalTrials.gov and received the ID NCT04333888.

**Supplementary Information:**

The online version contains supplementary material available at https://doi.org/10.1186/s12888-026-08767-7.

## Introduction

Borderline Personality Disorder (BPD) is characterized by intense emotional instability and impulsivity that has been linked to hyperactivity in the amygdala [1]. This is accompanied by disturbed amygdala connectivity within fronto-limbic networks, particularly with prefrontal and anterior cingulate regions, which are thought to support modulation of emotional responses [2]. These neural aberrations contribute to exaggerated affective responses and emotion dysregulation in BPD, as evidenced by consistent findings from fMRI (functional magnetic resonance imaging) studies and meta-analyses showing heightened amygdala activation to aversive stimuli [3, 4]. There are several evidence-based treatments for BPD recommended in clinical guidelines, including Dialectical Behavior Therapy (DBT), which has been shown to reduce amygdala hyperactivation in BPD, paralleling improvements in emotional symptoms [5, 6]. Although DBT is an evidence-based first-line treatment and can attenuate amygdala-related abnormalities, many patients show only partial remission [7, 8]. Moreover, individuals with BPD generally show persistent impairment despite symptom reduction and remain at risk for relapse [9].

Real-time functional magnetic resonance imaging (rt-fMRI) amygdala neurofeedback (amy-NF) enables participants to receive live feedback about activity in their amygdala and use that feedback to learn to modulate this activity. Compared to traditional psychotherapy treatments, amy-NF directly targets hyperactive amygdala responses [10]. Pilot amy-NF studies in BPD demonstrate sustained reductions in amygdala activation and BPD symptom severity after amy-NF training [11, 12]. Assuming that DBT and amy-NF act on the same neural circuit, amy-NF may be used to leverage psychotherapy when it is administered as an add-on to DBT. Recent research has increasingly focused on augmenting psychotherapy with pharmacological or neuromodulatory interventions with an aim to enhance the clinical and neuroplastic effects of psychotherapy [13, 14]. A recent review points to the emerging evidence base for combining neurofeedback with psychotherapeutic or related interventions, highlithing the need for further investigation [15]. Moreover, MacDuffie et al. found that one rt-fMRI neurofeedback session after cognitive-behavioral therapy increased the perceived efficacy and later use of the emotion-regulation strategies practiced during scanning, suggesting that neurofeedback may reinforce psychotherapy skills [16].

In this study, we aimed to study the additional value of combining amy-NF treatment with DBT. While prior amy-NF studies in BPD showed amygdala downregulation and improved emotion regulation, these studies lacked control groups and long-term follow-ups [11, 12]. The current study addressed these limitations by incorporating a treatment-as-usual (TAU) control arm and 3- and 6-month follow-up assessments. Building on results from the single-arm neurofeedback trial by Zaehringer at al., we used Ecological Momentary Assessment (EMA) to measure daily-life instability of negative affect as our primary endpoint [12]. The secondary endpoints included self-report measures and neural outcomes to further evaluate the clinical effect and neural mechanism of amy-NF, respectively. We recruited mid-therapy people with BPD with Borderline Symptom List–23 scores (BSL-23) [17] > 1.5 to address subjects with relatively high symptom severity after 6 weeks to the 12-week residential DBT program [18]. Given the resulting sample size, the study is better suited to providing preliminary evidence on the clinical and neural effects of the method than to a definitive assessment of efficacy. To our knowledge, this is the first study systematically combining amy-NF with DBT. We hypothesized that adding amy-NF to DBT would lead to greater reductions in daily-life affective instability compared with DBT alone.

## Methods

**Fig. 1:**
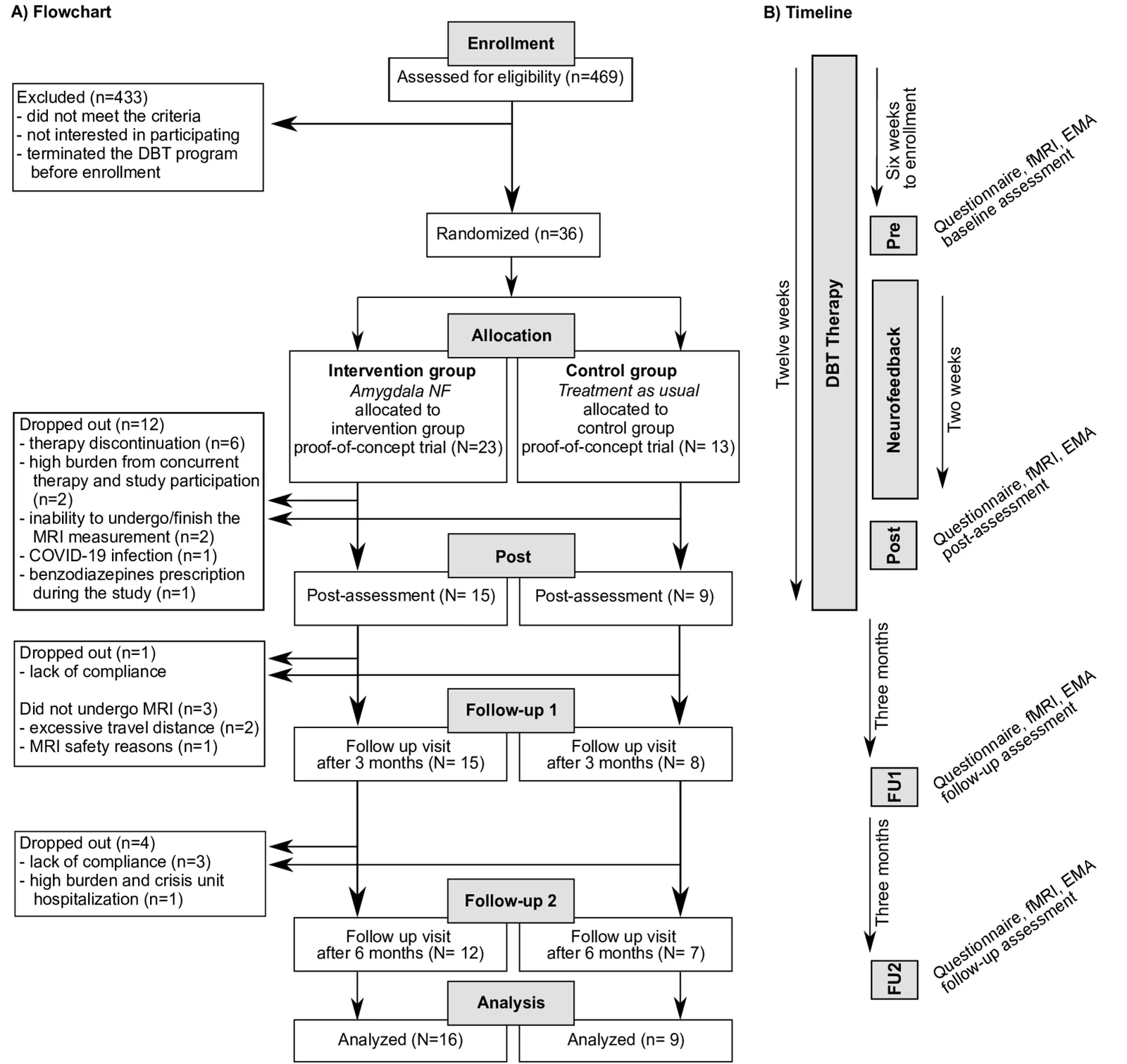
(**A**) Flowchart of subjects’ enrollment to the study. (**B**) Subjects’ timeline for study participation

Participants aged between 18 and 50 years undergoing a 12-week residential DBT program at the Department of Psychosomatic Medicine and Psychotherapy, Central Institute of Mental Health, were screened for eligibility between July 2020 and August 2023. Subjects were eligible if they fulfilled five or more of the Diagnostic and Statistical Manual of Mental Disorders-criteria for BPD [19], and their BSL-23 score was equal to or higher than 1.5 mid-therapy (i.e. after 6 weeks). The cutoff was originally 1.87, and was lowered to 1.5 during the study to support recruitment. Exclusion criteria were current pharmacotherapy with opioids, standing benzodiazepines (bedtime-only benzodiazepines and anti-histamines allowed), lifetime diagnosis of schizophrenia or bipolar disorder I, pregnancy, epilepsy, significant current or past neurological illness, BMI < 16.5, and usual safety criteria for magnetic resonance imaging. The participants were restricted from any substance use during the DBT program. The detailed flowchart of participant recruitment is depicted in Fig. 1A (cf. Supplement for details on randomization). The descriptive statistics of demographic and clinical variables are listed in Table 1. The participants in the NF group underwent 3 neurofeedback sessions each comprising 2 training runs. The timeline of all sessions and assessments is depicted in Fig. 1B.

**Table 1:** Sample demographics, baseline self-report measures and psychiatric characteristics

*N* (%)	NF		TAU		Total			
	23 (63.9)		13 (36.1)		36 (100)			
Female, N (%)	20 (87.0)		12 (92.3)		32 (88.9)			
Male, N (%)	3 (13)		1 (7.7)		4 (11.1)			
	**Mean**	**SD**	**Mean**	**SD**	**Mean**	**SD**	**U (n1**,** n2)**	**p**
Age	26.13	8.05	26.23	9.08	26.17	8.31	145 (13, 23)	0.895
Self-report measure								
Dissociative Experience Scale	31.4	14.26	24.38	14.43	28.83	14.49	74.5 (11, 19)	0.204
State-Trait Anxiety Inventory	68.37	5.02	63.64	14.81	66.63	9.83	99 (11, 19)	0.829
Childhood Trauma Questionnaire	72.63	14.83	54.36	16.51	65.93	17.62	47.5 (11, 19)	**0.015**
Difficulties in Emotion Regulation Scale	138.05	18.04	128.82	29.76	134.67	22.98	90 (11, 19)	0.546
Beck Depression Inventory II	40.00	6.23	35.82	15.49	38.47	10.54	98.5 (11, 19)	0.813
PTSD Checklist for DSM-5	71.89	9.75	61.64	16.97	68.13	13.55	69.5 (11, 19)	0.137
Barratt Impulsiveness Scale	76.21	10.23	71.45	7.27	74.47	9.42	76.5 (11, 19)	0.236
The Borderline Symptom List	2.59	0.72	2.15	1.07	2.43	0.87	81 (11, 19)	0.322
WHO Quality Of Life Questionnaire								
General	4.11	1.10	5.09	1.22	4.47	1.22	150.5 (11, 19)	**0.044**
Physical Health	40.41	14.58	48.05	20.6	43.21	17.10	129.5 (11, 19)	0.290
Psychological	17.76	10.83	23.86	18.55	20.00	14.16	122.5 (11, 19)	0.444
Social Relationships	40.79	19.22	50.00	20.07	44.17	19.71	130 (11, 19)	0.277
Environment	49.51	13.67	57.67	12.37	52.50	13.59	144 (11, 19)	0.092
Pittsburgh Sleep Quality Index	11.53	3.70	9.00	4.36	10.60	4.07	72.5 (11, 19)	0.171
Comorbidities	N	(%)	N	(%)	N	(%)		
Depressive disorders	22	(95.7)	13	(100)	35	(97.2)	-	1.000
Trauma-related disorders	14	(60.9)	3	(23.1)	17	(47.2)	-	**0.041**
Eating disorders	9	(39.1)	6	(46.2)	15	(41.7)	-	0.736
Substance use disorders	8	(34.8)	6	(46.2)	14	(38.9)	-	0.723
Anxiety / obsessive–compulsive disorders	5	(21.7)	3	(23.1)	8	(22.2)	-	1.000
Neurodevelopmental disorders	5	(21.7)	1	(7.7)	6	(16.7)	-	0.385
Other psychiatric disorders	6	(26.1)	4	(30.8)	10	(27.8)	-	1.000
Psychotropic medication								
SSRI	8	(34.8)	4	(30.8)	12	(33.3)	-	1.000
Serotonin antagonist	8	(34.8)	4	(30.8)	12	(33.3)	-	1.000
SNRI	6	(26.1)	7	(53.8)	13	(36.1)	-	0.150
Tetracyclic antidepressants	0	(0)	1	(7.7)	1	(2.8)	-	0.361
Tricyclic antidepressants	0	(0)	0	(0)	0	(0)	-	-
Other antidepressants	6	(26.1)	0	(0)	6	(16.7)	-	0.068
Conventional antipsychotica	3	(13)	2	(15.4)	5	(13.9)	-	1.000
Atypical antipsychotica	4	(17.4)	5	(38.5)	9	(25)	-	0.235
ADHD medication	4	(17.4)	0	(0)	4	(11.1)	-	0.274
Sleep medication	6	(26.1)	1	(7.7)	7	(19.4)	-	0.382
Other medication	9	(39.1)	5	(38.5)	14	(38.9)	-	1.000

Note: Mann-Whitney U test was used to test differences between groups in age and self-report measures, and Fisher’s exact test for differences in comorbidities and psychotropic medication. Diagnostic categories were defined based on ICD-10 codes: substance use disorders (F10.1–F19.1), depressive disorders (F32–F33), trauma-related disorders (F43), eating disorders (F50), anxiety/obsessive–compulsive disorders (F40–F42), neurodevelopmental disorders (F90, F95), and other psychiatric disorders (F44, F45, F60, F64)

NF = Neurofeedback, TAU = Treatment as usual, SSRI = Selective serotonin reuptake inhibitors, SNRI = Serotonin–norepinephrine reuptake inhibitor

The protocol was preregistered on ClinicalTrials.gov in April 2020 (https://clinicaltrials.gov/study/NCT04333888) [20], further details on preregistration and deviations are provided in the Supplement. Reporting followed the CONSORT 2025 and the CONSORT extension for randomized pilot and feasibility trials [21, 22].

### Assessments

#### Ecological momentary assessment

The pre-, post-, and 3-month-follow-up EMA lasted 4 days each and was scheduled to include two workdays and two weekend days or public holidays. EMA prompts were delivered via the movisensXS app (movisens GmbH, Karlsruhe, Germany) at randomized intervals, approximately once per hour between 9:00 a.m. and 10:00 p.m., with a minimum interval of 45 min, resulting in 13 prompts per day. Each prompt included 17 items assessing positive and negative affect, inner tension, dissociative symptoms (DSS-4), and positive and negative experiences. A full list of items is provided in the supplement.

#### Functional magnetic resonance imaging

Functional and structural MRI data were acquired at three timepoints: pre-, post-, and at the 3-month-follow-up session. All scans were conducted at the same 3T Siemens MAGNETOM Prisma Fit scanner using a 64-channel head coil. High-resolution structural images were acquired using a T1-weighted MPRAGE sequence (repetition time (TR) = 2 300 ms, echo time (TE) = 2.32 ms, inversion time (TI) = 900 ms, flip angle = 8°, voxel size = 0.9 × 0.9 × 0.9 mm³). Each session included two runs of resting-state fMRI (2 × 5 min; 150 volumes per run), an emotion regulation task (563 volumes), and an affective face-viewing task (370 volumes). All functional data were acquired using a T2*-weighted gradient echo-planar imaging (EPI) sequence with the following parameters: TR = 2 000 ms, TE = 30 ms, flip angle = 80°, 36 axial slices, slice thickness = 3 mm, field of view (FOV) = 192 × 192 mm², matrix size = 64 × 64, GRAPPA acceleration factor = 2.

The emotion regulation task consisted of the presentation of negative and neutral images. The task comprised three conditions: Regulate, View Negative, and View Neutral. In the Regulate condition, participants were instructed to regulate their emotional response to the image. In the View conditions, they were asked to simply view the image without trying to alter their emotional state. At the beginning of each trial, an instruction cue indicated the condition. Each trial lasted 12 s, during which the image was displayed on screen. Following the image presentation, participants rated their current level of emotional arousal using a 9-point SAM scale [23]. Trials were separated by an interstimulus interval with a fixation cross presented for 10–11.5 s.

During the faces task, participants viewed faces [24] displaying disgust, sadness, or fear (aversive condition) and scrambled face images (neutral condition) in a block design comprising 12 blocks per condition, with 6 stimuli per block. Each image was presented for 3 s (block duration: 18 s). Blocks were separated by an intertrial interval during which a fixation cross was shown for 12–13.5 s. To maintain attention, participants indicated the gender of the face in aversive blocks or the color of a surrounding frame in neutral blocks via button press.

During the resting state, participants were instructed to lay still and watch a white fixation cross on a dark grey background.

#### Self-report questionnaires

The questionnaire battery included measures of borderline symptom severity (BSL-23) [17], difficulties in emotion regulation (Difficulties in Emotion Regulation Scale; DERS) [25], depressive symptoms (Beck Depression Inventory–II; BDI-II) [26], trait impulsivity (Barratt Impulsiveness Scale–11; BIS-11) [27], posttraumatic stress symptoms (PTSD Checklist for DSM-5; PCL-5) [28], quality of life (WHOQOL-BREF) [29], and subjective sleep quality (Pittsburgh Sleep Quality Index; PSQI) [30].

### Neurofeedback training

The neurofeedback session consisted of two functional runs, acquired using a T2*-weighted EPI sequence (TR = 2 000 ms, TE = 30 ms, flip angle = 80°, voxel size = 3 × 3 × 3 mm³, 36 slices, GRAPPA acceleration factor = 2, 370 volumes per run).

**Fig. 2:**
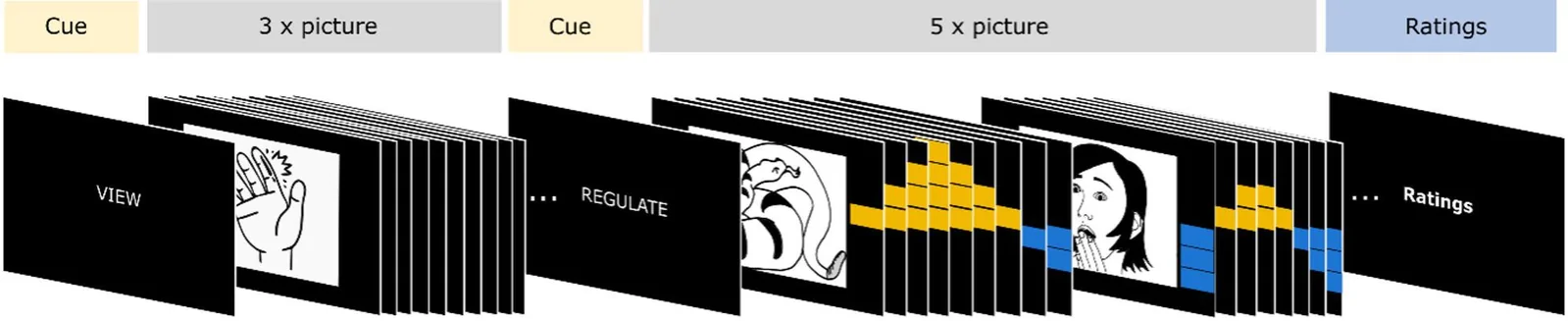
*Neurofeedback cycle*: Regulate blocks comprised five images, whereas View blocks comprised three images. In both conditions, each image was presented for 18 s. Participants provided ratings of emotional arousal and perceived regulation success at the end of each cycle

Functional images (TR = 2 s) were processed online using SPM12 and custom MATLAB scripts. The feedback signal was computed as the difference between right amygdala and control ROI activity and expressed as percent signal change. This signal was translated into an eight-level thermometer scale updated with each TR. The detailed description of the real-time analysis was previously published [31]. Each functional run comprised 4 cycles, during which participants were presented with negative images and instructed either to passively view the images (View condition) or to regulate the amygdala signal presented as feedback on both sides of the image, without receiving explicit strategy instructions (Regulate condition; Fig. 2).

Negative images for the emotion regulation and neurofeedback tasks were selected from multiple standardized affective image databases (IAPS [32], NAPS [33], EmoPicS [34], and OASIS [35]). All ratings were rescaled to a common 1–9 metric to ensure comparability. A total of 228 stimuli were selected and balanced (see Supplement for details) for arousal (minimum 4.46) and valence (maximum 4.76).

### Data processing and analysis

All statistical group comparisons were done in R software version 4.5.2 [36]. Unless stated otherwise, longitudinal outcomes were analyzed using linear mixed-effects models [37] with group, categorical time, and their interaction as fixed effects and random intercepts for subjects. TAU and pre-assessment were used as reference levels. Time was treated categorically to estimate changes across all assessments within a single longitudinal model, without assuming linear change across unequally spaced timepoints. Fixed effects were evaluated using Type III tests, and significant effects were followed up using Bonferroni-corrected estimated marginal means [38]. Further model-specific details are reported in the Supplement.

#### Ecological momentary assessment

EMA outcomes included negative affect, positive affect, inner tension, dissociative symptoms, and their instability. Affect scores were calculated by summing the respective positive and negative affect items, while dissociative symptoms were averaged across DSS-4 items [39]. Instability was quantified using the mean squared successive difference (MSSD) [40], calculated within participants and timepoints. Group differences over time were tested using mixed-effects models as described above; baseline group differences were inspected using estimated marginal means. Details on score calculation and model specifications are provided in the Supplement.

#### Functional magnetic resonance imaging

The fMRI data were converted to BIDS format [41] and preprocessed using fMRIPrep v20.0.6 [42]. Details on preprocessing, quality control, first-level models, and ROI extraction are provided in the Supplement. For task-based fMRI, amygdala activation was analyzed separately for the emotion regulation task and the faces task using BOLD contrast values as dependent variables: Regulate > View and Faces > Scrambled, respectively. Models included fixed effects of group, categorical time, region (left and right amygdala), and their interactions, with random intercepts for subjects. Although whole-brain analyses had been preregistered, analyses were restricted to amygdala ROIs because the available sample size was too small to yield reliable whole-brain results [43].

Subjective arousal ratings during the emotion regulation task were averaged within subject, timepoint, and condition. Regulation-related change was quantified as Regulate − View, with more negative values indicating greater reduction in subjective arousal, and analyzed using a mixed-effects model with group, categorical time, and their interaction as fixed effects.

Resting-state data were analyzed using the CONN toolbox [44] (details in Supplement). Seed-to-voxel analyses tested group differences in longitudinal changes in amygdala connectivity from pre- to post-assessment and from pre-assessment to 3-month follow-up using a bilateral amygdala seed.

#### Self-report questionnaires

In case of missing data, multiple imputation was applied to avoid further reductions in sample size (details in Supplement). Missingness was low, with no questionnaire exceeding 1%. For all questionnaire outcomes, total scores were analyzed using linear mixed-effects model [37] with group, categorical time, and their interaction as fixed effects and random intercepts for subjects. Time included pre-, post-, 3-month follow-up, and 6-month follow-up assessments. Details on imputation and model specifications are provided in the Supplement.

## Results

Participants showed evidence of successful downregulation during amy-NF training, as indicated by lower hemodynamic response function (HRF) amplitude and area under the curve (AUC) during Regulate compared with View trials. Full details of the HRF extraction and statistical analyses are provided in the Supplement.

### Ecological momentary assessment

**Fig. 3:**
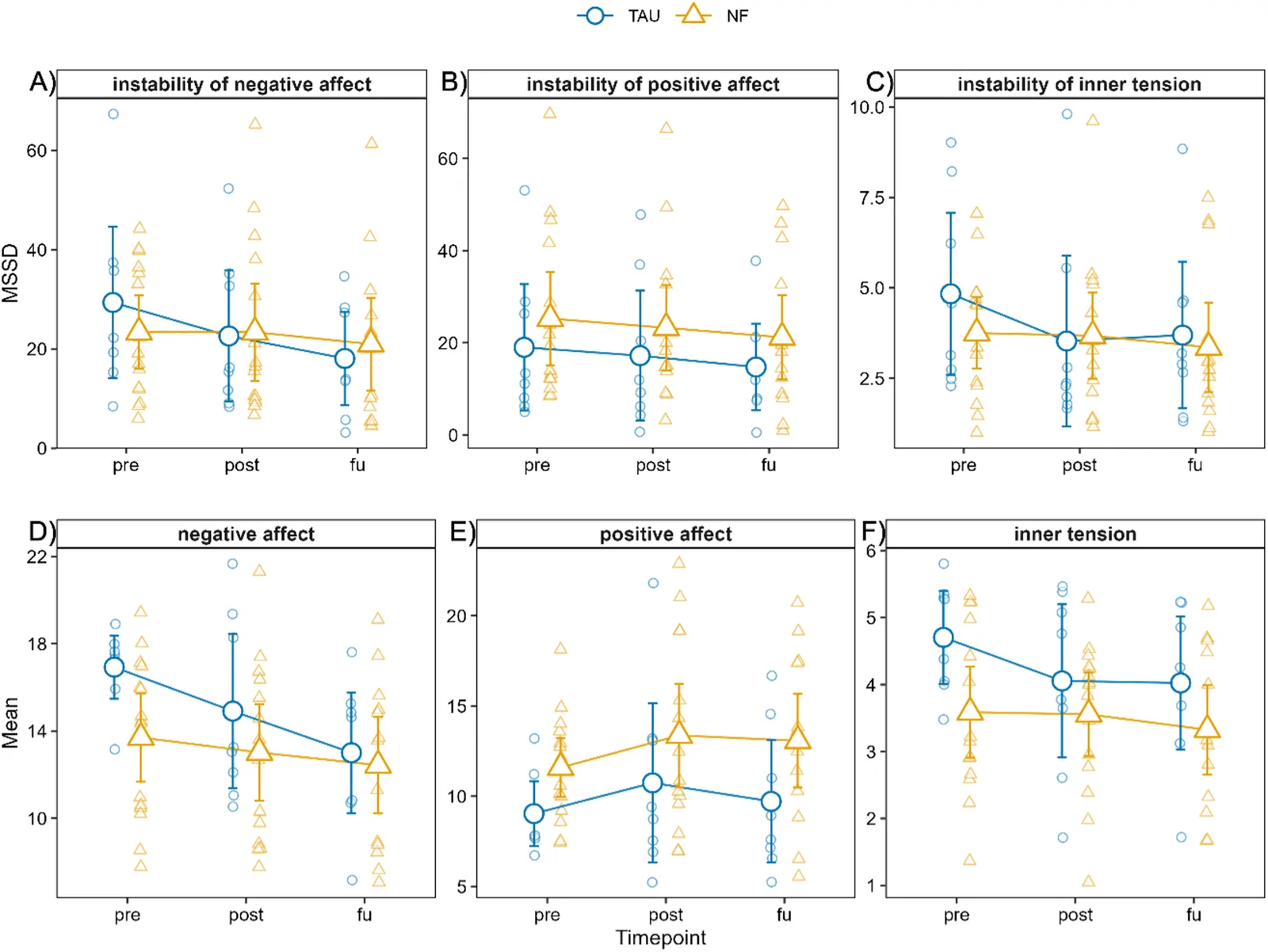
*Overview of EMA scores*: Mean EMA ratings for negative and positive affect (**D**-**F**), inner tension and their instability (MSSD; **A**-**C**) across pre-, post-, and follow-up assessments for TAU and NF groups. Individual data points show participant-level scores at each assessment. Error bars represent 95% confidence intervals

Compliance was high overall (M = 87.2%, SD = 9.1; range: 63.5–100%) and did not differ between groups, although it was lower at follow-up than at pre-assessment. Further details on compliance testing, and exploratory analyses of dissociative symptoms and reactivity to positive and negative experiences are provided in the Supplement. Model results for the main EMA outcomes are reported in Table 2 and depicted in Fig. 3.

**Table 2:** Statistical analyses of EMA outcomes

Outcome	Group	Time	Group x Time	Baseline/Significant post hoc	Interpretation
Instability of negative affect	F(1, 21.08) = 0.04, *p* = 0.848	F(2, 41.28) = 3.08, *p* = 0.056	F(2, 41.28) = 0.65, *p* = 0.526	Baseline: TAU > NF, *p* = 0.447	-
Instability of positive affect	F(1, 21.03) = 1.43, *p* = 0.245	F(2, 41.17) = 1.83, *p* = 0.173	F(2, 41.17) = 0.32, *p* = 0.724	Baseline: TAU < NF, *p* = 0.367	-
Instability of inner tension	F(1, 20.98) = 0.18, *p* = 0.679	F(2, 41.13) = 2.72, *p* = 0.078	F(2, 41.13) = 1.15, *p* = 0.327	Baseline: TAU > NF, *p* = 0.340	-
Negative affect	F(1, 21.01) = 1.51, *p* = 0.233	**F(2**,** 40.83) = 8.92**, *p***< 0.001**	F(2, 40.83) = 2.17, *p* = 0.127	Baseline: TAU > NF, *p* = 0.072; **post hoc: FU < pre**, *p***< 0.001**	Negative affect decreased over time.
Positive affect	F(1, 21.06) = 3.74, *p* = 0.067	F(2, 41.13) = 1.62, *p* = 0.210	F(2, 41.13) = 0.06, *p* = 0.938	Baseline: TAU < NF, *p* = 0.115	-
Inner tension	F(1, 20.98) = 3.02, *p* = 0.097	**F(2**,** 40.55) = 3.36**, *p*** = 0.045**	F(2, 40.55) = 1.27, *p* = 0.293	**Baseline: TAU > NF**, *p***= 0.035; post hoc: FU < pre**, *p*** = 0.046**	Inner tension decreased over time.

A simulation-based sensitivity analysis for the primary outcome (instability of negative affect), using the observed sample structure and variance components from the fitted mixed-effects model, indicated that 80% power (α = 0.05) was reached for a group × time interaction effect of approximately 1.7 residual standard deviations.

### Self-report questionnaires

Results for self-report questionnaires are reported in Table 3.

**Table 3:** Statistical analyses of self-report questionnaires

Outcome	Group	Time	Group x Time	Significant post hoc	Direction / interpretation
BSL-23 total	F(1, 35.81) = 0.87, *p* = 0.358	**F(3**,** 65.61) = 15.27**, *p* < 0.001	F(3, 65.61) = 0.16, *p* = 0.923	**Pre vs. 3-month/6-month FU**: **ts = -4.52 to -4.83**,** ps ≤ 0.0001** **Post vs. 3-month/6-month FU**: **ts = -4.86 to -5.17**,** ps < 0.0001**	BSL-23 scores increased from pre/post to both follow-ups, and the change did not differ between groups.
DERS total	F(1, 29.79) = 1.71, *p* = 0.201	**F(3**,** 60.08) = 4.00**, *p* = 0.012	F(3, 60.08) = 0.40, *p* = 0.756	**Pre vs. 3-month FU**: ***t*(62.9) = 3.18**, ***p*=0014**	Emotion dysregulation decreased over time and the change did not differ between groups. Post hoc tests indicated lower emotion dysregulation at 3-month follow-up than at pre-assessment.
BDI-II total	F(1, 32.66) = 1.83, *p* = 0.186	**F(3**,** 61.85) = 3.46**, *p* = 0.021	F(3, 61.85) = 0.70, *p* = 0.555	**Pre vs. post**: ***t*(63.7) = 2.85**, ***p*=0035**	In TAU, depressive symptom severity decreased over time and the change did not differ between groups. Post hoc tests indicated lower symptom severity at post-assessment than at pre-assessment.
BIS-11 total	F(1, 30.87) = 0.70, *p* = 0.408	F(3, 59.66) = 2.57, *p* = 0.063	F(3, 59.66) = 2.07, *p* = 0.114	None	No significant differences in impulsivity.
PCL-5 total	F(1, 30.63) = 3.20, *p* = 0.084	F(3, 62.88) = 1.28, *p* = 0.289	F(3, 62.88) = 0.78, *p* = 0.511	None	No significant differences in PTSD symptoms.
WHOQOL-BREF general QoL	F(1, 32.47) = 1.35, *p* = 0.254	F(3, 63.30) = 2.54, *p* = 0.065	F(3, 63.30) = 1.32, *p* = 0.277	None	No significant differences in general quality of life.
PSQI global	**F(1**,** 29.24) = 5.18**, *p* = 0.030	F(3, 59.78) = 1.46, *p* = 0.233	F(3, 59.78) = 0.08, *p* = 0.970	None	NF had generally poorer sleep quality than TAU.

### Functional magnetic resonance imaging

For the emotion regulation task, the Regulate > View contrast showed no significant group × time interaction, F(2, 105.63) = 2.86, *p* = 0.062, and no significant main effects of group or time. Subjective arousal ratings also did not differ significantly between groups or timepoints.

For the faces task, amygdala reactivity showed a significant group × time interaction, F(2, 94.17) = 7.07, *p* = 0.001, indicating differential change between groups. Post hoc comparisons showed that TAU decreased from pre- to post-assessment, *p* = 0.009, and increased from post-assessment to follow-up, *p* = 0.003, whereas changes in NF were not significant, all ps ≥ 0.084. At follow-up, amygdala reactivity was higher in TAU than in NF, *p* = 0.019, while no group differences were observed at pre- or post-assessment, both ps ≥ 0.391. Full model results for both tasks are reported in the Supplement.

For resting-state fMRI, seed-to-voxel analyses using the mean signal from the left and right amygdala as a bilateral seed showed greater pre-to-post connectivity change in NF relative to TAU in three significant clusters (*p* < 0.05 cluster-level FDR-corrected, cluster-defining threshold: *p* < 0.001 uncorrected). The largest cluster was centered in the precuneus and extended into the supplementary motor area, superior frontal gyrus, and postcentral gyrus (k = 234, pFDR < 0.001; peak MNI = [12], – [42, 52]). Two additional clusters were observed in superior/dorsomedial frontal regions (k = 104, pFDR = 0.006; peak MNI = [-10, -8, 78]) and (k = 70, pFDR = 0.030; peak MNI = [10, 12, 52]). No significant group differences were observed for the pre-to-follow-up contrast.

An exploratory analysis adjusting for baseline Childhood Trauma Questionnaires (CTQ) total score was conducted for all neural outcomes. The task results were unchanged, whereas in the resting state analysis, the significant effect was retained, although a larger number of significant clusters with a somewhat different spatial distribution was observed.

## Discussion

We investigated whether real-time fMRI amy-NF provides additional benefit when delivered additionally to residential DBT in people with BPD. Across groups, daily-life negative affect and inner tension decreased over time, indicating overall improvement during the study period. However, improvements were not larger in the NF group. While participants downregulated amygdala activity during training, the effects on daily-life emotional functioning, symptom measures and neural outcomes were mixed. Together, these findings suggest that amy-NF is feasible in this clinical setting and that voluntary regulation engages the neural target, i.e., the amygdala. However, the incremental benefit over DBT remains uncertain. Given the limited sample size and statistical power, the absence of significant between-group differences should not be interpreted as evidence of no additional effect, and the findings should be considered preliminary.

One possible explanation is that any additive effects of amy-NF were difficult to detect in the context of intensive residential DBT. DBT is associated with improvements in borderline-relevant outcomes [6], and neurobiological studies further suggest treatment-related changes in frontolimbic systems, including reduced amygdala responses following DBT [45]. In a design in which both groups receive a highly active intervention, any adjunctive effect must emerge over and above changes already induced by the background treatment. If DBT and amy-NF partly engage overlapping mechanisms of emotion regulation, the incremental contribution of NF may therefore be comparatively small and harder to distinguish statistically, particularly in a modest sample size. Consistent with this, the sensitivity analysis indicated that 80% power was reached only for group × time effects of about 1.7 residual standard deviations. Thus, smaller additive effects of amy-NF may have remained undetected. This interpretation remains speculative, but it represents one plausible explanation for why clear additive clinical benefits were not consistently observed.

At the level of daily-life experience, the EMA data suggest a general reduction in emotional distress across DBT treatment and follow-up, rather than a neurofeedback-specific effect. Contrary to our hypotheses, amy-NF was not associated with stronger reductions in affective instability or mean affective experience than TAU. Thus, the EMA findings do not provide evidence for an additional effect of amy-NF on daily-life affective dynamics beyond treatment as usual. Nevertheless, several findings point to improvement over time across groups. Mean levels of negative affect and inner tension decreased with significantly lower levels at 3-month follow-up than at pre-treatment. These findings are consistent with previously shown improvement in affective functioning during the inpatient DBT period, extending into follow-up [46, 47]. The absence of significant pre-to-post changes, together with significant reductions at follow-up, may indicate that changes in daily-life emotional distress unfold gradually and become more apparent after treatment completion. This delayed pattern is consistent with the possibility that skills acquired during DBT require time to be transferred into everyday emotional functioning.

Several factors complicate interpretation of the EMA findings. First, repeated EMA prompting may itself have influenced affective reporting by increasing attention to emotional states [48]. As the same EMA protocol was applied in both groups, such reactivity would be more likely to affect overall time trends than differential treatment effects; however, EMA-related measurement reactivity was not assessed directly. Second, Interpretation of group differences in EMA outcomes must take into account baseline disparities between groups. Across some EMA measures, the NF group exhibited lower baseline distress, reflected in lower inner tension and marginally lower negative affect, which may have limited the potential for further improvement on the outcome, thereby reducing the likelihood of detecting a stronger decline over time (see Fig. 3). More generally, the small sample size and limited degrees of freedom may have reduced statistical power to detect modest group × time effects.

Self-report measures showed modest changes over time that were not specific to neurofeedback (see Figure S3 and Figure S4). Borderline symptom severity increased at follow-up in both groups, indicating a general worsening after discharge without a differential intervention effect. This increase contrasts with the delayed reductions in daily-life negative affect and inner tension observed in the EMA data, suggesting that the gradual transfer of DBT skills into everyday emotional functioning may not have translated into improvements in perceived borderline-related symptom burden after discharge. Depressive symptoms showed a significant decrease at post-assessment across groups, consistent with overall clinical improvement during residential treatment, with no group-specific differences over time. DERS scores also decreased significantly over time, with lower emotion dysregulation at follow-up. Other self-report measures showed nonsignificant fluctuations or isolated effects, without a consistent pattern favoring neurofeedback.

Participants successfully downregulated amygdala activity during neurofeedback, particularly in the early training runs (see Supplement), consistent with previous studies [12, 49]. Downregulation was strongest during the first four runs, replicating the temporal pattern reported by Zaehringer et al. [12], whereas condition differences diminished in the two additional runs, possibly reflecting habituation to repeated exposure to negative affective stimuli.

In the emotion regulation task, Regulate > View contrast showed no neurofeedback-related reduction, indicating no evidence that NF selectively enhanced deliberate emotion regulation. However, visual inspection of the individual conditions suggested that amygdala responses during both Regulate and View decreased from pre- to post-assessment in the NF group but increased in TAU (Figure S6A), pointing to broader changes in amygdala engagement rather than selective enhancement of regulation. These effects were not maintained at follow-up and may reflect transient habituation to repeated negative stimuli during neurofeedback training. Although improved amygdala habituation to repeated unpleasant pictures have been reported following DBT in BPD [6], the increase in amygdala responses from pre- to post-assessment in the TAU group suggests that this pattern was specific to the NF group, potentially due to the additional exposure during neurofeedback training.

In the faces task, amygdala reactivity to emotionally salient social stimuli decreased from pre- to post-assessment in TAU but increased again by follow-up, whereas NF showed no significant change, despite a descriptive reduction from pre-assessment to follow-up. Consequently, amygdala reactivity was higher in TAU than NF at follow-up, with no group differences at earlier timepoints. This may indicate a treatment-related reduction in TAU that was not maintained after discharge, and a more stable NF trajectory, although the absence of significant within-group change in NF limits interpretation.

Amygdala reactivity to emotional stimuli tended to decrease over the course of treatment in prior DBT neuroimaging studies [45]. However, in those studies, neural changes appeared more closely coupled with improvements in self-reported emotion regulation and symptom severity [6], which was not evident in the present study. Moreover, previous rt-fMRI amygdala neurofeedback studies have shown task-related amygdala modulation [11] and EMA-based affective improvements [12], but without control groups and with limited effects on broader emotion regulation outcomes. Evidence from other clinical populations suggests that neural target engagement and clinical effects may vary across disorders. In depression, amygdala neurofeedback during positive autobiographical recall has been associated with successful amygdala modulation and symptom improvement [50], whereas in posttraumatic stress disorder, improved amygdala control did not translate into significantly greater symptom reduction [51]. More broadly, a meta-analysis of amygdala neurofeedback studies supports the feasibility of learned amygdala modulation but highlights limited evidence linking such modulation to behavioral and clinical effects [10]. Thus, neurofeedback may modify amygdala responses without necessarily yielding robust clinical benefits when added to effective standard treatment.

Relative to TAU, NF was associated with a transient pre-to-post increase in resting-state connectivity between the bilateral amygdala and precuneus/dorsomedial frontal regions. This aligns with work implicating amygdala–precuneus coupling in emotional processing and modulation, specifically attentional deployment during emotion regulation [52]. Fan et al. further showed that altered amygdala–precuneus connectivity is related to childhood trauma in depression [53], underscoring the relevance of this pathway for affective vulnerability. Childhood maltreatment has also been associated with altered neural responses to emotional stimuli, including heightened amygdala reactivity [54]. Given the higher CTQ scores in the NF group, childhood trauma severity may therefore have influenced neural responses and their change over time. In exploratory analyses adjusting for baseline CTQ total score, the task-fMRI findings remained unchanged, while the resting-state NF > TAU pre-to-post connectivity effect persisted but showed a somewhat different spatial distribution. Thus, the observed neural differences cannot be attributed to baseline trauma severity alone, but the small sample and substantial CTQ imbalance limit firm conclusions about the specific contribution of neurofeedback. Above that, prior amy-NF work in BPD reported connectivity changes mainly with lateral prefrontal regions [11]. Together with the task-based results, our findings therefore suggest a transient shift in amygdala coupling with midline systems, pointing to altered affective processing rather than selective enhancement of voluntary regulation.

Across outcomes, the present findings provide limited evidence for convergence between neural, daily-life, and self-report measures. While neurofeedback was associated with some changes in amygdala responses, these changes did not consistently correspond with changes in daily-life affect dynamics or self-reported symptoms. Importantly, against the background of strong clinical improvement during residential DBT, any added benefit of neurofeedback may have been comparatively small and therefore difficult to detect. This is consistent with the current state of clinical rt-fMRI neurofeedback research [10, 49], where reliable target engagement is often demonstrated, but translation into generalized clinical outcomes is variable. Accordingly, recent trial designs proposals [10, 55, 56] increasingly emphasize methodological specificity, such as the inclusion of sham control conditions, as well as clinically meaningful everyday-life endpoints.

The findings also raise questions about optimal timing. During residential DBT, patients were already engaged in intensive treatment and learning multiple emotion-regulation skills, which may have limited the added impact of neurofeedback. The post-discharge increase in borderline symptoms in both groups highlights the challenge of maintaining gains during care transitions, consistent with known unstable symptom trajectories in BPD [9]. Neurofeedback may therefore be more useful after discharge, when patients can apply it to consolidate and strengthen previously acquired regulation strategies [16]. Future studies should test whether neurofeedback is more effective as a maintenance or booster intervention than as concurrent augmentation during intensive treatment.

### Limitations

Several limitations should be considered. First, recruitment was challenging, limiting sample size and statistical power. The originally planned sample size (22/group) was not reached due to strict inclusion/exclusion criteria, including symptom severity and clinical stability requirements, and disruptions related to the COVID-19 pandemic. The possibility of Type II error therefore cannot be excluded, and the findings should be interpreted within the context of a pilot randomized controlled trial rather than as a definitive test of efficacy. The small sample also limits generalizability and precluded more fine-grained analyses, such as linking neural changes to clinical outcomes. Above that, the allocation ratio was changed from 1:1 to 60:40 in favor of neurofeedback during the study (see Supplement for details). The resulting group imbalance may have modestly reduced the precision of between-group estimates.

Second, baseline differences in several EMA and self-report measures complicated interpretation. Despite randomization, these imbalances likely reflect sampling variability in a small clinical sample and warrant cautious interpretation of differential trajectories.

Third, neurofeedback was delivered alongside residential DBT without a sham neurofeedback control. Although clinically realistic, this limits causal attribution to neurofeedback, and the intensity of concurrent DBT may have constrained detection of additive clinical effects.

Finally, assessment timing may have affected findings. Baseline assessments occurred mid-treatment, whereas follow-up took place after discharge, a period of substantial contextual change that may have contributed to symptom fluctuations and obscured intervention-specific effects.

## Conclusions

Real-time fMRI amygdala neurofeedback was feasible in a highly symptomatic residential DBT population and engaged the amygdala during training. However, within the constraints of this small sample, the study did not demonstrate consistent added benefits over treatment as usual for daily-life affect dynamics or self-reported symptoms, and neural effects were not regulation-specific. Thus, although amygdala neurofeedback can modulate neural responses, its incremental clinical benefit during residential DBT remains uncertain. Future studies should test whether neurofeedback is more effective later in treatment or after discharge to support maintenance of therapeutic gains.

## Supplementary Information

Below is the link to the electronic supplementary material.

Supplementary Material 1

## Data Availability

Analysis code and data are available on the Open Science Framework: https://osf.io/cet7j/. Analysis code is openly available, while data are available via controlled access.

## Abbreviations

amy-NF: Amygdala neurofeedback
AUC: Area under the curve
BDI-II: Beck Depression Inventory-II
BIS-11: Barratt Impulsiveness Scale-11
BOLD: Blood-oxygen-level-dependent
BPD: Borderline personality disorder
BSL-23: Borderline Symptom List-23
CTQ: Childhood Trauma Questionnaire
DBT: Dialectical behavior therapy
DERS: Difficulties in Emotion Regulation Scale
DSS-4: Dissociation-Tension Scale-4
EMA: Ecological momentary assessment
EPI: Echo-planar imaging
fMRI: Functional magnetic resonance imaging
HRF: Hemodynamic response function
MSSD: Mean squared successive difference
NF: Neurofeedback
PCL-5: PTSD Checklist for DSM-5
PSQI: Pittsburgh Sleep Quality Index
PTSD: Post-traumatic stress disorder
ROI: Region of interest
rt-fMRI: Real-time functional magnetic resonance imaging
TAU: Treatment as usual
TR: Repetition time
WHOQOL-BREF: World Health Organization Quality of Life–BREF
