# Supplementary material for: Amygdala neurofeedback as an adjunct to residential dialectical behavior therapy for borderline personality disorder: a pilot randomized controlled trial
Source: BMC Psychiatry. 2026 Oct 16;26:802. doi: 10.1186/s12888-026-08767-7 (PMC13675997; doi:10.1186/s12888-026-08767-7)
Supplement: Supplementary file 1 [file 12888_2026_8767_MOESM1_ESM.docx]

Supplementary Material

Contents

[Methods details	2](#_Toc241595050)

[Preregistered outcomes and deviations	2](#_Toc241595051)

[Randomization	2](#_Toc241595052)

[Ecological Momentary Assessment	2](#_Toc241595053)

[Compliance	2](#_Toc241595054)

[EMA items and score calculation	3](#_Toc241595055)

[Statistical models	5](#_Toc241595056)

[Functional Magnetic Resonance Imaging	6](#_Toc241595057)

[Balancing affective stimuli	6](#_Toc241595058)

[Preprocessing of fMRI data using fMRIPrep	6](#_Toc241595059)

[Task fMRI analysis	8](#_Toc241595060)

[Resting-state data preprocessing and analysis	9](#_Toc241595061)

[Self-reports (data imputiation)	10](#_Toc241595062)

[Supplementary figures and results	11](#_Toc241595063)

[Effect sizes for main study outcomes	11](#_Toc241595064)

[Ecological Momentary Assessment	13](#_Toc241595065)

[Response rate (figure)	13](#_Toc241595066)

[Dissociative symptoms and reactivity to positive and negative experiences (exploratory results)	13](#_Toc241595067)

[Self-report questionnaires (figures)	16](#_Toc241595068)

[Functional Magnetic Resonance Imaging	17](#_Toc241595069)

[Neurofeedback Training (results)	17](#_Toc241595070)

[Emotion Regulation and Faces task (results)	18](#_Toc241595071)

[References	19](#_Toc241595072)

# Methods details

## Preregistered outcomes and deviations

The preregistered primary outcome was EMA-based instability of negative affect. Preregistered secondary outcomes comprised BSL-23 symptom severity; amygdala BOLD activity for the Regulate > View contrast in the emotion-regulation task; whole-brain BOLD effects for the same contrast; resting-state amygdala connectivity; and amygdala BOLD activity for the Faces > Scrambled contrast in the faces task. The preregistered whole-brain analysis of the Regulate > View contrast was not performed because the final sample size was considered insufficient for a reliable whole-brain analysis. Diffusion tensor imaging (DTI) measures of white-matter microstructure were preregistered as exploratory outcomes but were not analyzed because scanner-related technical problems rendered these data unusable.

## Randomization

Randomization was based on stratified permuted blocks with two strata (sex and clinic). Several measures were taken to ensure concealed assignment. First, randomization was performed by an independent statistician while all persons involved in the recruitment process were kept blind until written informed consent was provided by the participant. Second, in order to avoid that the pending group assignments can be derived from previous assignments, blocks were of variable length, and the length of blocks was unknown to all persons involved in the recruitment. Randomization initially used a 1:1 allocation ratio. Because recruitment was slower than anticipated and the planned sample size was unlikely to be reached, the ratio was changed to 60:40 in favor of neurofeedback for the final 11 participants. This was done to increase the number receiving the experimental intervention and thereby obtain more information on its clinical and neural effects. The change was independent of outcomes observed in the existing sample. No minimization was used beyond stratification by sex and clinic.

## Ecological Momentary Assessment

### Compliance

Compliance was calculated as the percentage of completed prompts (out of 13 prompts per day) for each subject and timepoint. Participants with a response rate below 50% were excluded from further analyses (n = 1). The final sample included n = 8 participants in the TAU group and n = 15 (14 at follow-up) in the NF group. To examine potential differences in compliance across assessment timepoints between groups, we fitted a mixed-effects model[1] with fixed effects of group (TAU, NF), time (pre, post, 3-month follow-up), and their interaction, and a random intercept for subject. Pairwise comparisons between timepoints were performed using estimated marginal means (emmeans package in R) [2] with Bonferroni correction.

The model showed no significant group × time interaction, F(2, 41.17) = 0.47, p = .629. There was also no significant main effect of group, F(1, 20.95) = 0.40, p = .531. However, there was a significant main effect of time, F(2, 41.17) = 8.55, p < .001. Bonferroni-corrected post hoc comparisons indicated that compliance was lower at follow-up than at pre, p < .001, whereas differences between pre and post, p = .080, and between post and follow-up, p = .218, were not significant. Plotted compliance rates are depicted in the Supplement (Figure S2).

### EMA items and score calculation

All EMA items are listed in Supplementary Table S1. Negative affect was calculated for each inquiry by summing the five negative affect items: sad, irritated, angry, depressed, and anxious. Positive affect was calculated by summing the five positive affect items: happy, relaxed, content, cheerful, and enthusiastic. The DSS-4 score was calculated as the average of four items assessing dissociative symptoms. To quantify the instability of positive affect, negative affect, inner tension, and dissociative symptoms, squared successive differences were calculated. For each participant, timepoint, and day, differences between consecutive scores were computed and squared, resulting in squared successive difference values. The mean squared successive difference was then calculated by averaging these values within each participant and day. Daily mean squared successive difference values were subsequently averaged across the respective timepoint, yielding one instability score per participant and timepoint for each EMA domain.

**Table S1** EMA items

| Assessed variables | Item | Scale |
| --- | --- | --- |
| Positive affect | Right now, I feel happy.  *(Im Moment fühle ich mich glücklich.)* | 1 - 7 |
|  | Right now, I feel relaxed.  *(Im Moment fühle ich mich entspannt).* | 1 - 7 |
|  | Right now, I feel content.  *(Im Moment fühle ich mich zufrieden.)* | 1 - 7 |
|  | Right now, I feel excited/cheerful.  *(Im Moment fühle ich mich fröhlich.)* | 1 - 7 |
|  | Right now, I feel enthusiastic.  *(Im Moment fühle ich mich enthusiastisch.)* | 1 - 7 |
| Negative affect | Right now, I feel sad.  *(Im Moment fühle ich mich traurig.)* | 1 - 7 |
|  | Right now, I feel irritated.  *(Im Moment fühle ich mich gereizt.)* | 1 - 7 |
|  | Right now, I feel angry.  *(Im Moment fühle ich mich wütend.)* | 1 - 7 |
|  | Right now, I feel depressed.  *(Im Moment fühle ich mich niedergeschlagen.)* | 1 - 7 |
|  | Right now, I feel anxious.  *(Im Moment fühle ich mich ängstlich.)* | 1 - 7 |
| Inner tension | Right now, I feel unpleasant inner tension.  *(Im Moment stehe ich unter unangenehmer innerer Anspannung.)* | 0 - 9 |
| DSS-4 | At the moment, I have the impression that my body doesn’t belong to me.  *(Im Moment habe ich die Empfindung als ob mein Körper nicht zu mir gehört.)* | 0 - 9 |
|  | At the moment, I have problems hearing; e.g. I hear sounds from nearby as if they come from far away.  *(Im Moment habe ich* *Probleme richtig zu hören, z.B. höre ich die Geräusche um mich herum, als ob sie von weit weg kommen.)* | 0 - 9 |
|  | At the moment, I have the impression other people or things around me are unreal.  *(Im Moment habe ich die Empfindung, andere Menschen oder andere Dinge oder die Welt um mich herum seien nicht wirklich.)* | 0 - 9 |
|  | At the moment, I have the impression that my body or parts of it are insensitive to pain.  *(Im Moment habe ich die Empfindung, dass mein Körper oder einzelne Teile davon unempfindlich gegenüber körperlichen Schmerzen sind.)* | 0 - 9 |
| Reactivity to positive and negative experiences | Have you had one or more bad experiences since the last survey? How bad was your worst experience?  *(Hattest Du seit der letzten Abfrage eines oder mehrere schlechte Erlebnisse? Wie schlimm war Dein schlechtestes Erlebnis?)* | 1 - 7 |
|  | Have you had one or more good experiences since the last survey? How good was your best experience?  *(Hattest Du seit der letzten Abfrage eines oder mehrere gute Erlebnisse? Wie gut war Dein bestes Erlebnis?)* | 1 - 7 |

### Statistical models

For EMA outcomes, mixed-effects models were fitted with group, categorical time, and their interaction as fixed effects. Group was coded as TAU and NF, with TAU as the reference group. Time was modeled as a categorical factor with three levels: pre-assessment, post-assessment, and 3-month follow-up, with pre-assessment as the reference level. Fixed effects were evaluated using Type III tests. Significant effects were followed up using estimated marginal means with Bonferroni-corrected pairwise comparisons. Baseline group differences were also inspected using estimated marginal means.

Log-transformed *MSSD* scores (log(x + 1)) for *positive and negative affect, DSS-4, and inner tension* were analyzed using linear mixed-effects models estimated with REML for subject with random intercepts. Fixed effects were tested using Type III ANOVA with Satterthwaite’s approximation of degrees of freedom.

Positive and negative affect scores were log-transformed using log(x + 1) to reduce skewness and normalize their distributions before analysis. Linear mixed-effects models estimated with restricted maximum likelihood (REML) were fitted for *positive and negative affect,* and *inner tension*. These models included random intercepts for subject and for subject-by-time to account for repeated observations within each subject and timepoint. Fixed effects were tested using Type III ANOVA with Satterthwaite’s approximation of degrees of freedom.

For *DSS-4* scores, a two-part generalized mixed-effects approach was applied to account for the large proportion of zero values and the non-normal distribution of symptom severity. First, a binary presence model with a logit link tested whether dissociative symptoms were present. Second, among non-zero observations, symptom severity was modeled using log-transformed symptom scores with a Student-*t* error distribution. Both models included random intercepts for subjects and subject-by-time clusters to account for repeated EMA observations. Fixed effects were tested using Type III Wald tests.

*Reactivity to negative experiences* and *reactivity to positive experiences* were analyzed using a one-inflated two-part approach. First, logistic mixed-effects models with a logit link estimated the probability of no negative experience, defined as scores of 1. Second, among responses greater than 1, ordinal intensity was modeled using cumulative link mixed models with a logit link for scores 2–7. Both the binary and ordinal models included subject-level and subject-by-time random intercepts. Fixed effects were tested using Type III Wald tests.

Model assumptions were assessed using residual diagnostics appropriate to each model type, including residual plots for mixed models and DHARMa diagnostics for generalized models. For ordinal models, the proportional odds assumption was additionally checked.

## Functional Magnetic Resonance Imaging

### Balancing affective stimuli

First, arousal ratings were balanced across the three emotion regulation runs (pre-, post-, and follow-up 1) and six neurofeedback runs (ANOVA, p > .9). After assigning the first 60 images to the emotion regulation runs, the remaining images were re-balanced across neurofeedback blocks. Finally, the order of images within neurofeedback blocks was adjusted such that View trials contained images with medium arousal levels, ensuring consistent within-block variability.

### Preprocessing of fMRI data using fMRIPrep

Results included in this manuscript come from preprocessing performed using *fMRIPrep* 20.0.6[4, 5]; RRID:SCR_016216), which is based on *Nipype* 1.4.2[6, 7]; RRID:SCR_002502).

**Anatomical data preprocessing**

A total of 3 T1-weighted (T1w) images were found within the input BIDS dataset. All of them were corrected for intensity non-uniformity (INU) with N4BiasFieldCorrection[8], distributed with ANTs 2.2.0 ([9], RRID:SCR_004757). The T1w-reference was then skull-stripped with a *Nipype* implementation of the antsBrainExtraction.sh workflow (from ANTs), using OASIS30ANTs as target template. Brain tissue segmentation of cerebrospinal fluid (CSF), white-matter (WM) and gray-matter (GM) was performed on the brain-extracted T1w using fast (FSL 5.0.9, RRID:SCR_002823,[10]). A T1w-reference map was computed after registration of 3 T1w images (after INU-correction) using mri_robust_template (FreeSurfer 6.0.1,[11]). Brain surfaces were reconstructed using recon-all (FreeSurfer 6.0.1, RRID:SCR_001847,[12]), and the brain mask estimated previously was refined with a custom variation of the method to reconcile ANTs-derived and FreeSurfer-derived segmentations of the cortical gray-matter of Mindboggle (RRID:SCR_002438,[13]). Volume-based spatial normalization to one standard space (MNI152NLin2009cAsym) was performed through nonlinear registration with antsRegistration (ANTs 2.2.0), using brain-extracted versions of both T1w reference and the T1w template. The following template was selected for spatial normalization: *ICBM 152 Nonlinear Asymmetrical template version 2009c* [[14], RRID:SCR_008796; TemplateFlow ID: MNI152NLin2009cAsym],

**Functional data preprocessing**

For each of the 12 BOLD runs found per subject (across all tasks and sessions), the following preprocessing was performed. First, a reference volume and its skull-stripped version were generated using a custom methodology of *fMRIPrep*. A B0-nonuniformity map (or *fieldmap*) was estimated based on a phase-difference map calculated with a dual-echo GRE (gradient-recall echo) sequence, processed with a custom workflow of *SDCFlows* inspired by the [epidewarp.fsl script](http://www.nmr.mgh.harvard.edu/~greve/fbirn/b0/epidewarp.fsl) and further improvements in HCP Pipelines[15]. The *fieldmap* was then co-registered to the target EPI (echo-planar imaging) reference run and converted to a displacements field map (amenable to registration tools such as ANTs) with FSL’s fugue and other *SDCflows* tools. Based on the estimated susceptibility distortion, a corrected EPI (echo-planar imaging) reference was calculated for a more accurate co-registration with the anatomical reference. The BOLD reference was then co-registered to the T1w reference using bbregister (FreeSurfer) which implements boundary-based registration[16]. Co-registration was configured with six degrees of freedom. Head-motion parameters with respect to the BOLD reference (transformation matrices, and six corresponding rotation and translation parameters) are estimated before any spatiotemporal filtering using mcflirt (FSL 5.0.9,[17]). BOLD runs were slice-time corrected using 3dTshift from AFNI 20160207 ([18], RRID:SCR_005927). The BOLD time-series (including slice-timing correction when applied) were resampled onto their original, native space by applying a single, composite transform to correct for head-motion and susceptibility distortions. These resampled BOLD time-series will be referred to as *preprocessed BOLD in original space*, or just *preprocessed BOLD*. The BOLD time-series were resampled into standard space, generating a *preprocessed BOLD run in MNI152NLin2009cAsym space*. First, a reference volume and its skull-stripped version were generated using a custom methodology of *fMRIPrep*. Several confounding time-series were calculated based on the *preprocessed BOLD*: framewise displacement (FD), DVARS and three region-wise global signals. FD and DVARS are calculated for each functional run, both using their implementations in *Nipype* (following the definitions by Power et al.[19]). The three global signals are extracted within the CSF, the WM, and the whole-brain masks. Additionally, a set of physiological regressors were extracted to allow for component-based noise correction (*CompCor*,[20]). Principal components are estimated after high-pass filtering the *preprocessed BOLD* time-series (using a discrete cosine filter with 128s cut-off) for the two *CompCor* variants: temporal (tCompCor) and anatomical (aCompCor). tCompCor components are then calculated from the top 5% variable voxels within a mask covering the subcortical regions. This subcortical mask is obtained by heavily eroding the brain mask, which ensures it does not include cortical GM regions. For aCompCor, components are calculated within the intersection of the aforementioned mask and the union of CSF and WM masks calculated in T1w space, after their projection to the native space of each functional run (using the inverse BOLD-to-T1w transformation). Components are also calculated separately within the WM and CSF masks. For each CompCor decomposition, the *k* components with the largest singular values are retained, such that the retained components’ time series are sufficient to explain 50 percent of variance across the nuisance mask (CSF, WM, combined, or temporal). The remaining components are dropped from consideration. The head-motion estimates calculated in the correction step were also placed within the corresponding confounds file. The confound time series derived from head motion estimates and global signals were expanded with the inclusion of temporal derivatives and quadratic terms for each[21]. Frames that exceeded a threshold of 0.5 mm FD or 1.5 standardised DVARS were annotated as motion outliers. All resamplings can be performed with *a single interpolation step* by composing all the pertinent transformations (i.e. head-motion transform matrices, susceptibility distortion correction when available, and co-registrations to anatomical and output spaces). Gridded (volumetric) resamplings were performed using antsApplyTransforms (ANTs), configured with Lanczos interpolation to minimize the smoothing effects of other kernels[22]. Non-gridded (surface) resamplings were performed using mri_vol2surf (FreeSurfer).

Many internal operations of *fMRIPrep* use *Nilearn* 0.6.2 ([23], RRID:SCR_001362), mostly within the functional processing workflow. For more details of the pipeline, see [the section corresponding to workflows in *fMRIPrep*’s documentation](https://fmriprep.readthedocs.io/en/latest/workflows.html).

### Task fMRI analysis

All preprocessed data were visually inspected for quality, including functional alignment, and data exhibiting excessive head motion (maximum framewise displacement ≥ 4 mm) were excluded from further analyses. Preprocessed functional images were spatially smoothed with a 6 mm full-width at half-maximum Gaussian kernel.

First-level statistical models for the emotion regulation task and the faces task were implemented in SPM12 using a custom MATLAB script[24]. The design matrix for each subject included regressors of interest derived from condition-specific stimulus onset times, convolved with SPM’s canonical hemodynamic response function. Six motion parameters (three translations, three rotations) from realignment were added as nuisance regressors, and high-pass filtering (128 s cutoff) was applied to remove low-frequency drifts. Contrast images were generated for task-specific comparisons for each subject.

ROI-based analyses were conducted using the MarsBaR toolbox[25] (version 0.44) for SPM. Subject-level first-level models served as the basis for ROI extraction. Binary ROI masks of the left and right amygdala were applied to extract mean contrast estimates for each subject and contrast of interest. These extracted values were used for subsequent statistical analyses.

For both fMRI tasks, data from 27 participants (NF = 17, TAU = 10) were available for statistical analysis at pre-assessment. At post-assessment, data were available from 22 participants for the faces task (NF = 14, TAU = 8) and 23 participants for the emotion regulation task (NF = 14, TAU = 9). At 3-month follow-up, data were available from 16 participants for the faces task (NF = 11, TAU = 5) and 17 participants for the emotion regulation task (NF = 12, TAU = 5).

#### Neurofeedback Training (offline analysis)

ROI time series were extracted from first-level models computing the eigenvariate within the right amygdala mask used for neurofeedback training and adjusting for the effects-of-interest. Extracted BOLD signals were used to estimate condition-specific hemodynamic response functions (HRF) for each picture presentation using an inverse logit model [3]. From this model, HRF amplitude and area under the curve (AUC) parameters were derived.

HRF amplitude and log-transformed AUC were analyzed using linear models with condition, run, and their interaction as fixed effects (AUC was log-transformed to improve model diagnostics). Run was modeled continuously from 1 to 6, allowing the condition × run interaction to test whether linear change across runs differed by condition. Significant interactions were followed up by estimating the linear effect of run within each condition using estimated marginal trends. Condition differences at each run were examined using estimated marginal means, with Bonferroni correction across the six run-specific comparisons. Sample size varied across runs (n = 15–20 participants per run) due to drop-out and data exclusion.

### Resting-state data preprocessing and analysis

Analyses of fMRI-resting-state data were performed using CONN[26] (RRID:SCR_009550) release 22.v2407[27] and SPM[28] (RRID:SCR_007037) release 12.7771.

**Preprocessing**: FMRIPrep-preprocessed functional and anatomical data were further preprocessed using a modular preprocessing pipeline[29] including removal of initial scans, outlier detection, and smoothing. The first 5 scans in each functional run were removed. Potential outlier scans were identified using ART[30] as acquisitions with framewise displacement above 0.9 mm or global BOLD signal changes above 5 standard deviations[19, 31], and a reference BOLD image was computed for each subject by averaging all scans excluding outliers. Last, functional data were smoothed using spatial convolution with a Gaussian kernel of 6 mm full width half maximum (FWHM).

**Denoising**: In addition, functional data were denoised using a standard denoising pipeline[29] including the regression of potential confounding effects characterized by white matter timeseries (5 CompCor noise components), CSF timeseries (5 CompCor noise components), motion parameters and their first order derivatives (12 factors)[32], outlier scans (below 22 factors)[19], session and task effects and their first order derivatives (12 factors), and linear trends (2 factors) within each functional run, followed by bandpass frequency filtering of the BOLD timeseries[33] between 0.008 Hz and 0.09 Hz. CompCor[20, 34] noise components within white matter and CSF were estimated by computing the average BOLD signal as well as the largest principal components orthogonal to the BOLD average, motion parameters, and outlier scans within each subject's eroded segmentation masks. From the number of noise terms included in this denoising strategy, the effective degrees of freedom of the BOLD signal after denoising were estimated to range from 61 to 214.5 (average 167.6) across all subjects[31].

**First-level analysis**: Seed-based connectivity maps were estimated characterizing the patterns of functional connectivity with atlas.Amygdala r, and atlas.Amygdala l (right and left amygdala derived from the Harvard-Oxford Cortical atlas). Functional connectivity strength was represented by Fisher-transformed bivariate correlation coefficients from a weighted general linear model (weighted-GLM[29]), defined separately for each pair of seed and target areas, modeling the association between their BOLD signal timeseries. Individual scans were weighted by a boxcar signal characterizing each individual task or experimental condition convolved with an SPM canonical hemodynamic response function and rectified.

**Group-level analyses** were performed using a General Linear Model (GLM[29]). For each individual voxel a separate GLM was estimated, with first-level connectivity measures at this voxel as dependent variables (one independent sample per subject and one measurement per timepoint), and groups or other subject-level identifiers as independent variables. Voxel-level hypotheses were evaluated using multivariate parametric statistics with random-effects across subjects and sample covariance estimation across multiple measurements. Inferences were performed at the level of individual clusters (groups of contiguous voxels). Cluster-level inferences were based on parametric statistics from Gaussian Random Field theory[29, 35]. Results were thresholded using a combination of a cluster-forming p < 0.001 voxel-level threshold, and a familywise corrected p-FDR < 0.05 cluster-size threshold[36].

Resting-state data from 30 participants (NF = 20, TAU = 10) were available for analysis, with data from 23 participants (NF = 14, TAU = 9) at post-treatment and 18 (NF = 13, TAU = 5) at 3-month follow-up.

## Self-reports (data imputiation)

Item-level imputation was conducted using multiple imputation by chained equations with predictive mean matching (20 imputations) in the mice package in R[37], restricted to theoretically defined item sets (subscales or full item sets, depending on the questionnaire) and including subject, group, and timepoint as auxiliary predictors.

# Supplementary figures and results

## Effect sizes for main study outcomes

**Table S2** Descriptive statistics and effect sizes for main study outcomes

|  | NF: pre / post / FU1 / FU2 | | TAU: pre / post / FU1 / FU2 | | Between group effect size: Hedge’s g [95% CI] | | |
| --- | --- | --- | --- | --- | --- | --- | --- |
| Outcome | Mean (SD) | N | Mean (SD) | N | Pre-Post | Pre-FU1 | Pre-FU2 |
| EMA | | | | | | | |
| Instability of negative affect | 23.4 / 23.4 / 21.0 / -  (13.3 / 17.7 / 16.2 / -) | 15 / 15 / 14 / - | 29.4 / 22.6 / 18.1 / -  (18.3 / 15.8 / 11.2 / -) | 8 / 8 / 8 / - | 0.42  [−0.42, 1.25] | 0.43  [−0.42, 1.27] | - |
| Instability of positive affect | 25.3 / 23.3 / 21.2 / -  (18.3 / 16.7 / 15.8 / -) |  | 19.0 / 17.2 / 14.7 / -  (16.4 / 16.9 / 11.2 / -) |  | −0.01  [−0.84, 0.82] | −0.04  [−0.87, 0.80] | - |
| Instability of inner tension | 3.75 / 3.68 / 3.36 / -  (1.78 / 2.15 / 2.15 / -) |  | 4.84 / 3.53 / 3.69 / -  (2.68 / 2.82 / 2.42 / -) |  | 0.57  [−0.28, 1.41] | 0.33  [−0.52, 1.17] | - |
| Negative affect | 13.7 / 13.1 / 12.6 / -  (3.64 / 4.02 / 3.97 / -) |  | 17.0 / 14.8 / 12.9 / -  (1.74 / 4.36 / 3.26 / -) |  | 0.50  [−0.35, 1.33] | 1.01  [0.10, 1.89] | - |
| Positive affect | 11.6 / 13.2 / 13.0 / -  (2.95 / 4.89 / 4.34 / -) |  | 9.00 / 10.8 / 9.79 / -  (2.17 / 5.55 / 4.08 / -) |  | −0.06  [−0.89, 0.77] | 0.13  [−0.71, 0.97] | - |
| Inner tension | 3.60 / 3.57 / 3.38 / -  (1.24 / 1.07 / 1.13 / -) |  | 4.73 / 4.05 / 4.01 / -  (0.83 / 1.43 / 1.20 / -) |  | 0.76  [−0.11, 1.61] | 0.39  [−0.46, 1.23] | - |
| Self-reports | | | | | | | |
| BSL-23 | 2.48 / 2.37 / 3.21 / 3.13  (0.69 / 0.81 / 0.74 / 0.79) | 23 / 15 / 15 / 12 | 2.22 / 2.22 / 3.04 / 2.98  (1.05 / 1.01 / 1.10 / 0.91) | 12 / 8 / 8 / 7 | 0.04  [−0.79, 0.87] | −0.21  [−1.07, 0.66] | −0.18  [−1.11, 0.76] |
| DERS total | 138 / 130 / 121 / 123  (18 / 16.9 / 19.4 / 18.6) | 19 / 16 / 15 / 12 | 129 / 121 / 122 / 121  (29.8 / 30.4 / 36.6 / 26.8) | 11 / 9 / 8 / 7 | 0.39  [−0.46, 1.22] | −0.10  [−0.98, 0.78] | −0.07  [−1.03, 0.88] |
| BDI-II total | 40 / 36.1 / 34 / 33.8  (6.23 / 7.43 / 8.86 / 11) |  | 35.8 / 27.9 / 30.1 / 25.1  (15.5 / 20 / 24.2 / 17.6) |  | 0.31  [−0.53, 1.15] | −0.38  [−1.26, 0.51] | −0.03  [−0.99, 0.93] |
| BIS-11 total | 76.2 / 71.1 / 72.1 / 73.3  (10.2 / 9.33 / 10.7 / 9.99) |  | 71.5 / 70.1 / 74.6 / 67.7  (7.27 / 9.85 / 11.1 / 7.50) |  | −0.21  [−1.05, 0.63] | −0.81  [−1.72, 0.12] | 0.13  [−0.83, 1.09] |
| PCL-5 total | 71.9 / 68.2 / 67.7 / 64.8  (9.75 / 9.10 / 10.7 / 11.6) |  | 61.6 / 58 / 63.4 / 55.9  (17 / 18.1 / 19.6 / 25) |  | −0.09  [−0.93, 0.75] | −0.69  [−1.59, 0.22] | −0.39  [−1.35, 0.59] |
| WHOQOL-BREF general QoL | 4.11 / 5.12 / 5 / 5.08  (1.10 / 0.89 / 1.81 / 1.31) |  | 5.09 / 5.44 / 5.5 / 5.14  (1.22 / 1.24 / 1.41 / 1.21) |  | 0.63  [−0.24, 1.48] | 0.22  [−0.67, 1.10] | 0.89  [−0.13, 1.89] |
| PSQI global | 11.5 / 11.1 / 10.9 / 9.92  (3.70 / 3.47 / 2.42 / 3.18) |  | 9 / 8.56 / 8.12 / 6.43  (4.36 / 4.72 / 3.64 / 3.41) |  | 0.42  [−0.43, 1.26] | −0.06  [−0.94, 0.82] | −0.05  [−1.00, 0.91] |
| fMRI task contrasts (amygdala) | | | | | | | |
| Emotion Regulation (left) | −0.26 / −0.20 / −0.06 / -  (0.46 / 0.30 / 0.57 / -) | 17 / 14 / 12 / - | 0.00 / 0.16 / −0.06 / -  (0.54 / 0.30 / 0.25 / -) | 10 / 9 / 5 / - | −0.07  [−0.89, 0.75] | 0.73  [−0.33, 1.77] | - |
| Emotion Regulation (right) | −0.13 / −0.18 / 0.02 / -  (0.35 / 0.30 / 0.66 / -) |  | −0.02 / 0.04 / −0.16 / -  (0.49 / 0.20 / 0.19 / -) |  | −0.04  [−0.86, 0.78] | 0.80  [−0.27, 1.84] | - |
| Faces (left) | 0.63 / 0.49 / 0.30 / -  (0.35 / 0.38 / 0.56 / -) | 17 / 14 / 11 / - | 0.77 / 0.58 / 0.91 / -  (0.43 / 0.21 / 0.39 / -) | 10 / 8 / 5 / - | 0.80  [−0.10, 1.67] | −0.57  [−1.59, 0.48] | - |
| Faces (right) | 0.72 / 0.60 / 0.48 / -  (0.35 / 0.42 / 0.47 / -) |  | 0.81 / 0.64 / 0.97 / -  (0.53 / 0.13 / 0.40 / -) |  | 0.64  [−0.24, 1.50] | −0.45  [−1.46, 0.58] | - |

Note: Descriptive statistics and standardized between-group effect sizes (Hedges’ g, NF − TAU, with 95% confidence intervals) are reported on the original scale. Where required, inferential mixed-effects models were fitted to transformed outcomes to satisfy model assumptions.

## Ecological Momentary Assessment

### Response rate (figure)

**Figure S1** Boxplots of EMA response rates by timepoint and group. Each box represents the distribution of individual participants’ response rates (in %), with whiskers indicating variability across subjects.

### Dissociative symptoms and reactivity to positive and negative experiences (exploratory results)

In the symptom presence model for *DSS-4* scores, the group × time interaction was not significant, χ²(2) = 0.89, p = .641. The main effects of time, χ²(2) = 0.99, p = .610, and group, χ²(1) = 0.19, p = .665, were also not significant. Similarly, in the symptom severity model, the group × time interaction was not significant, χ²(2) = 0.34, p = .844, and the main effects of time, χ²(2) = 0.37, p = .831, and group, χ²(1) = 0.00, p = .978, were not significant. Model diagnostics indicated deviations from uniformity, while dispersion and outlier tests were not significant.

For *instability of dissociative symptoms* (MSSD), the group × time interaction was not significant, F(2, 41.03) = 0.75, p = .480, indicating that the change in instability of dissociative symptoms over time did not differ between groups. There was a significant main effect of time, F(2, 41.03) = 3.84, p = .030, whereas the main effect of group was not significant, F(1, 20.96) = 0.45, p = .511. Bonferroni-corrected post-hoc comparisons indicated significantly lower instability at post-treatment than at pre-treatment, p = .027, whereas all other contrasts were not significant (p > .298).

The following exploratory EMA items assessed whether participants had experienced one or more positive or negative experiences since the previous survey (e.g., “Have you had one or more good/bad experiences since the last survey?”) and, if so, the intensity of the most salient experience (e.g., “How bad was your best/worst experience?”). Responses ranged from 1 (no experience) to 7 (highest intensity); binary models contrasted no experience (1) with any reported experience (2–7), while ordinal models examined intensity of the experience across ratings 2–7.

In the binary model for *reactivity to negative experiences*, the group × time interaction was significant, χ²(2) = 10.00, p = .007. The main effect of time was also significant, χ²(2) = 17.48, p < .001, whereas the main effect of group was not, χ²(1) = 0.10, p = .754. Follow-up contrasts indicated that TAU had a higher probability of reporting no negative experience than NF at follow-up, p = .024, but not at pre- or post-treatment. Within-group contrasts showed that the probability of reporting no negative experience increased significantly from pre-treatment to follow-up (p < .001) and from post-treatment to follow-up (p = .006) in the TAU group, whereas no significant time differences were observed in the NF group, all ps ≥ .787.

In the ordinal model, the group × time interaction was not significant, χ²(2) = 2.03, p = .363. There was a significant main effect of time, χ²(2) = 7.08, p = .029, indicating decreasing intensity of negative experiences over time, whereas the main effect of group was not significant, χ²(1) = 0.53, p = .466. Bonferroni-corrected comparisons indicated generally lower intensity at follow-up than at pre-treatment, p = .005.

In the binary model for *reactivity to positive experiences*, the group × time interaction was not significant, χ²(2) = 1.41, p = .493. The main effects of time, χ²(2) = 2.42, p = .299, and group, χ²(1) = 0.77, p = .379, were also not significant.

In the ordinal model, the group × time interaction was not significant, χ²(2) = 3.40, p = .183. The main effects of time, χ²(2) = 2.77, p = .250, and group, χ²(1) = 1.23, p = .267, were also not significant. Because the proportional-odds assumption was not fully supported for this ordinal component, results were evaluated against a log-transformed mixed-effects sensitivity model (using the full response scale 1-7). This model yielded the same substantive conclusion, with no significant main or interaction effects (all ps > .089), suggesting that reactivity to positive experiences remained stable across timepoints in both groups. Reactivity to both positive and negative experiences is depicted in Figure S3.

**Figure S2** *Reactivity to positive and negative experiences (EMA)* across timepoints (pre, post, follow-up) and groups. Panels display mean overall rating (A), the probability of reporting any experience (B), and mean intensity of the reported experiences (C). Error bars represent 95% confidence intervals.

## Self-report questionnaires (figures)

**Figure S3** *Borderline Symptom List–23:* Mean BSL-23 score across pre-, post-, 3-month-follow-up (FU1) and 6-month-follow-up (FU2) assessments for TAU and NF groups. Individual data points show participants‘ scores and error bars represent 95% confidence intervals.

**Figure S4** *Self-report questionnaires:* Total scores of Difficulties in Emotion Regulation Scale (A), Beck Depression Inventory–II (B), Barratt Impulsiveness Scale–11 (C), PTSD Checklist for DSM‑5 (D), Quality of life measured with WHOQOL-BREF (general items only) (E), Pittsburgh Sleep Quality Index (F) across pre-, post-, 3-month-follow-up (FU1), and 6-month-follow-up (FU2) assessments for TAU and NF groups. Higher scores indicate greater difficulties, except for WHOQOL-BREF illustrating the opposite direction. Error bars represent 95% confidence intervals.

## Functional Magnetic Resonance Imaging

### Neurofeedback Training (results)

A linear model for HRF amplitude revealed a significant condition × run interaction, F(1, 200) = 5.36, p = .022. The main effect of condition was significant, F(1, 200) = 8.30, p = .004, while the main effect of run was not, F(1, 200) = 2.21, p = .138. Follow-up analyses showed that amplitude significantly decreased across runs during viewing (b = −0.087, SE = 0.033, p = .008), but did not change significantly during downregulation (b = 0.019, SE = 0.033, p = .559). Bonferroni-adjusted comparisons showed higher amplitude during viewing than downregulation in runs 1–3 (ps ≤ .007), but not in runs 4–6 (ps ≥ .353).

For log-transformed AUC, there was a significant condition × run interaction, F(1, 200) = 9.11, p = .003. There were also significant main effects of condition, F(1, 200) = 14.76, p < .001, and run, F(1, 200) = 4.97, p = .027. Follow-up analyses showed that AUC significantly decreased across runs during viewing (b = −0.125, SE = 0.034, p < .001), but did not change significantly during downregulation (b = 0.019, SE = 0.034, p = .577). Bonferroni-adjusted comparisons showed higher AUC during viewing than downregulation in runs 1–3 (ps ≤ .0001), but not in runs 4–6 (ps ≥ .068). Residual diagnostics were improved by log-transforming AUC, although some left-tail deviations remained; results should therefore be interpreted with caution.

**Figure S5** *Amygdala amplitude and AUC for Regulate and View condition during neurofeedback training*. A) The amygdala amplitude was significantly reduced comparing the Regulate vs. View condition during the first three training runs. B) AUC was significantly reduced comparing the Regulate and View condition during the first four training runs. Error bars represent 95% confidence intervals.

### Emotion Regulation and Faces task (results)

**Table S3** Model results for Emotion Regulation and Faces task

| **Task** | **Group** | **Time** | **Region** | **Group × Time** | **Region-related effects** | **Post hoc comparisons** |
| --- | --- | --- | --- | --- | --- | --- |
| Emotion Regulation | F(1, 22.10) = 0.86,  p = .363 | F(2, 105.63) = 0.30,  p = .738 | F(1, 92.04) = 0.002,  p = .967 | F(2, 105.63) = 2.86,  p = .062 | all ps ≥ .287 | n/a |
| Faces | F(1, 23.91) = 1.09,  p = .307 | **F(2, 94.17) = 4.34,**  **p = .016** | F(1, 89.29) = 2.84,  p = .095 | **F(2, 94.17) = 7.07,**  **p = .001** | all ps ≥ .468 | **TAU: pre > post, p = .009; post < FU, p = .003;**  **TAU > NF at FU, p = .019;**  NF: pre > FU p = .084 |

**Figure S6** *Emotion Regulation Task and Faces Task:* Lines represent group means (TAU, NF) across timepoints (pre, post, follow-up), shaded areas indicate 95% confidence intervals. A) Emotion Regulation Task: Mean contrast estimates for the Regulate, View (View Negative), and Neutral (View Neutral) conditions relative to the implicit baseline in the left amygdala. Results for the right amygdala showed a similar pattern. B) Emotion Regulation Task: Group-averaged contrast estimates for the Regulate vs. View contrast in the left and right amygdala. C) Faces Task: Group-averaged contrast estimates for the faces vs. scrambled contrast in the left and right amygdala.

## References

1. Bates D, Mächler M, Bolker B, Walker S. Fitting Linear Mixed-Effects Models Using **lme4**. J Stat Softw. 2015;67. https://doi.org/10.18637/jss.v067.i01.

2. Searle SR, Speed FM, Milliken GA. Population Marginal Means in the Linear Model: An Alternative to Least Squares Means. Am Stat. 1980;34:216–21. https://doi.org/10.1080/00031305.1980.10483031.

3. Lindquist MA, Wager TD. Validity and power in hemodynamic response modeling: a comparison study and a new approach. Hum Brain Mapp. 2007;28:764–84. https://doi.org/10.1002/hbm.20310.

4. Esteban O, Markiewicz CJ, DuPre E, Goncalves M, Kent JD, Ciric R, et al. fMRIPrep: a robust preprocessing pipeline for functional MRI. 2020. https://doi.org/10.5281/ZENODO.3754722.

5. Esteban O, Markiewicz CJ, Blair RW, Moodie CA, Isik AI, Erramuzpe A, et al. fMRIPrep: a robust preprocessing pipeline for functional MRI. Nat Methods. 2019;16:111–6. https://doi.org/10.1038/s41592-018-0235-4.

6. Gorgolewski K, Burns CD, Madison C, Clark D, Halchenko YO, Waskom ML, et al. Nipype: A Flexible, Lightweight and Extensible Neuroimaging Data Processing Framework in Python. Front Neuroinformatics. 2011;5. https://doi.org/10.3389/fninf.2011.00013.

7. Gorgolewski KJ, Esteban O, Markiewicz CJ, Burns C, Goncalves M, Jarecka D, et al. nipy/nipype: 1.11.0. 2026. https://doi.org/10.5281/ZENODO.596855.

8. Tustison NJ, Avants BB, Cook PA, Yuanjie Zheng, Egan A, Yushkevich PA, et al. N4ITK: Improved N3 Bias Correction. IEEE Trans Med Imaging. 2010;29:1310–20. https://doi.org/10.1109/TMI.2010.2046908.

9. Avants B, Epstein C, Grossman M, Gee J. Symmetric diffeomorphic image registration with cross-correlation: Evaluating automated labeling of elderly and neurodegenerative brain. Med Image Anal. 2008;12:26–41. https://doi.org/10.1016/j.media.2007.06.004.

10. Zhang Y, Brady M, Smith S. Segmentation of brain MR images through a hidden Markov random field model and the expectation-maximization algorithm. IEEE Trans Med Imaging. 2001;20:45–57. https://doi.org/10.1109/42.906424.

11. Reuter M, Rosas HD, Fischl B. Highly accurate inverse consistent registration: A robust approach. NeuroImage. 2010;53:1181–96. https://doi.org/10.1016/j.neuroimage.2010.07.020.

12. Dale AM, Fischl B, Sereno MI. Cortical Surface-Based Analysis. NeuroImage. 1999;9:179–94. https://doi.org/10.1006/nimg.1998.0395.

13. Klein A, Ghosh SS, Bao FS, Giard J, Häme Y, Stavsky E, et al. Mindboggling morphometry of human brains. PLOS Comput Biol. 2017;13:e1005350. https://doi.org/10.1371/journal.pcbi.1005350.

14. Fonov V, Evans A, McKinstry R, Almli C, Collins D. Unbiased nonlinear average age-appropriate brain templates from birth to adulthood. NeuroImage. 2009;47:S102. https://doi.org/10.1016/S1053-8119(09)70884-5.

15. Glasser MF, Sotiropoulos SN, Wilson JA, Coalson TS, Fischl B, Andersson JL, et al. The minimal preprocessing pipelines for the Human Connectome Project. NeuroImage. 2013;80:105–24. https://doi.org/10.1016/j.neuroimage.2013.04.127.

16. Greve DN, Fischl B. Accurate and robust brain image alignment using boundary-based registration. NeuroImage. 2009;48:63–72. https://doi.org/10.1016/j.neuroimage.2009.06.060.

17. Jenkinson M, Bannister P, Brady M, Smith S. Improved Optimization for the Robust and Accurate Linear Registration and Motion Correction of Brain Images. NeuroImage. 2002;17:825–41. https://doi.org/10.1006/nimg.2002.1132.

18. Cox RW, Hyde JS. Software tools for analysis and visualization of fMRI data. NMR Biomed. 1997;10:171–8. https://doi.org/10.1002/(SICI)1099-1492(199706/08)10:4/5%3C171::AID-NBM453%3E3.0.CO;2-L.

19. Power JD, Mitra A, Laumann TO, Snyder AZ, Schlaggar BL, Petersen SE. Methods to detect, characterize, and remove motion artifact in resting state fMRI. NeuroImage. 2014;84:320–41. https://doi.org/10.1016/j.neuroimage.2013.08.048.

20. Behzadi Y, Restom K, Liau J, Liu TT. A component based noise correction method (CompCor) for BOLD and perfusion based fMRI. NeuroImage. 2007;37:90–101. https://doi.org/10.1016/j.neuroimage.2007.04.042.

21. Satterthwaite TD, Elliott MA, Gerraty RT, Ruparel K, Loughead J, Calkins ME, et al. An improved framework for confound regression and filtering for control of motion artifact in the preprocessing of resting-state functional connectivity data. NeuroImage. 2013;64:240–56. https://doi.org/10.1016/j.neuroimage.2012.08.052.

22. Lanczos C. Evaluation of Noisy Data. J Soc Ind Appl Math Ser B Numer Anal. 1964;1:76–85. https://doi.org/10.1137/0701007.

23. Abraham A, Pedregosa F, Eickenberg M, Gervais P, Mueller A, Kossaifi J, et al. Machine learning for neuroimaging with scikit-learn. Front Neuroinformatics. 2014;8. https://doi.org/10.3389/fninf.2014.00014.

24. Paret C. christianparet/RaBIDS: v.0.3.1. 2023. https://doi.org/10.5281/ZENODO.4738347.

25. Brett M, Anton J-L, Valabregue R, Poline J-B. Region of Interest Analysis Using an SPM Toolbox. Neuroimage. 2002;16. https://doi.org/10.1016/S1053-8119(02)90013-3.

26. Whitfield-Gabrieli S, Nieto-Castanon A. *Conn* : A Functional Connectivity Toolbox for Correlated and Anticorrelated Brain Networks. Brain Connect. 2012;2:125–41. https://doi.org/10.1089/brain.2012.0073.

27. Nieto-Castanon A, Whitfield-Gabrieli S. CONN functional connectivity toolbox: RRID SCR_009550, release 22. 22nd edition. Hilbert Press; 2022. https://doi.org/10.56441/hilbertpress.2246.5840.

28. Penny WD, Friston KJ, Ashburner J, Kiebel S, Nichols T, editors. Statistical parametric mapping: the analysis of funtional brain images. First edition. Amsterdam Boston: Elsevier/Academic Press; 2007.

29. Nieto-Castanon A. Handbook of functional connectivity magnetic resonance imaging methods in CONN. Boston, MA: Hilbert Press; 2020.

30. Whitfield-Gabrieli S, Nieto-Castanon A, Ghosh S. Artifact Detection Tools (ART). 2011.

31. Nieto-Castanon A. Preparing fMRI Data for Statistical Analysis. 2022. https://doi.org/10.48550/ARXIV.2210.13564.

32. Friston KJ, Williams S, Howard R, Frackowiak RS, Turner R. Movement-related effects in fMRI time-series. Magn Reson Med. 1996;35:346–55. https://doi.org/10.1002/mrm.1910350312.

33. Hallquist MN, Hwang K, Luna B. The nuisance of nuisance regression: spectral misspecification in a common approach to resting-state fMRI preprocessing reintroduces noise and obscures functional connectivity. NeuroImage. 2013;82:208–25. https://doi.org/10.1016/j.neuroimage.2013.05.116.

34. Chai XJ, Castañón AN, Ongür D, Whitfield-Gabrieli S. Anticorrelations in resting state networks without global signal regression. NeuroImage. 2012;59:1420–8. https://doi.org/10.1016/j.neuroimage.2011.08.048.

35. Worsley KJ, Marrett S, Neelin P, Vandal AC, Friston KJ, Evans AC. A unified statistical approach for determining significant signals in images of cerebral activation. Hum Brain Mapp. 1996;4:58–73. https://doi.org/10.1002/(SICI)1097-0193(1996)4:1%3C58::AID-HBM4%3E3.0.CO;2-O.

36. Chumbley J, Worsley K, Flandin G, Friston K. Topological FDR for neuroimaging. NeuroImage. 2010;49:3057–64. https://doi.org/10.1016/j.neuroimage.2009.10.090.

37. Buuren SV, Groothuis-Oudshoorn K. **mice** : Multivariate Imputation by Chained Equations in *R*. J Stat Softw. 2011;45. https://doi.org/10.18637/jss.v045.i03.
